# L-Arginine and Intermittent Hypoxia Are Stress-Limiting Factors in Male Wistar Rat Models

**DOI:** 10.3390/ijms252212364

**Published:** 2024-11-18

**Authors:** Natalia Kurhaluk, Oleksandr Lukash, Piotr Kamiński, Halina Tkaczenko

**Affiliations:** 1 Department of Animal Physiology, Institute of Biology, Pomeranian University in Słupsk, Arciszewski St. 22b, PL 76-200 Słupsk, Poland; halina.tkaczenko@upsl.edu.pl; 2 Department of Ecology, Geography and Nature Management, T.H. Shevchenko National University “Chernihiv Colehium”, Hetmana Polubotka St. 53, 14013 Chernihiv, Ukraine; lukash2011@ukr.net; 3 Department of Medical Biology and Biochemistry, Collegium Medicum in Bydgoszcz, Nicolaus Copernicus University in Toruń, M. Skłodowska-Curie St. 9, PL 85-094 Bydgoszcz, Poland; piotr.kaminski@cm.uml.pl; 4 Department of Ecology and Environmental Protection, Collegium Medicum in Bydgoszcz, Nicolaus Copernicus University in Toruń, M. Skłodowska-Curie St. 9, PL 85-094 Bydgoszcz, Poland; 5 Department of Biotechnology, Faculty of Biological Sciences, Institute of Biological Sciences, University of Zielona Góra, Prof. Z. Szafran St. 1, PL 65-516 Zielona Góra, Poland

**Keywords:** intermittent hypoxia training, L-arginine, N^ω^-nitro-L-arginine (L-NNA), oxidative stress, oxygen-dependent processes, mitochondrial respiration

## Abstract

The aim of this study was to evaluate the combined effects of L-arginine, intermittent hypoxia training (IHT), and acute stress on oxygen-dependent processes in rats, including mitochondrial oxidative phosphorylation, microsomal oxidation, and the intensity of lipoperoxidation processes. In addition, our study investigated how the modulatory effect of the NO synthase mechanism on the concentration of catecholamines (CA), such as adrenaline and noradrenaline, and their biosynthetic precursors (DOPA, dopamine) varies depending on the cholinergic (acetylcholine, Ach-acetylcholinesterase, AChE) status in rats. This study investigated the protective stress-limiting effects of L-arginine impact and IHT in the blood and liver of rats. The results showed that L-arginine promoted the maintenance of NAD-dependent oxidation in mitochondria, which was detrimental compared to succinate oxidation, and was accompanied by depletion of respiratory activity reserves under stress induced by high concentrations of CA. The interdependence of SC-dependent oxidation and the functional role of NAD-dependent substrate oxidation in the mitochondrial respiratory chain in stress conditions induced using inhibitors revealed the importance of the NO system. Administration of L-arginine during the IHT course prior to stress exposure increased the compensatory capacity of the organism. L-arginine increased the compensatory capacity of the sympathoadrenal system in stress-exposed rats. In the early stages of IHT, modulation of the CA concentration was observed with a concomitant increase in lipoperoxidation processes, and in the final stages of IHT, the CA concentrations increased, but there was also an inhibition of lipoperoxidation, which was particularly enhanced by the administration of L-arginine. The increase in blood concentrations of CA and ACh was accompanied by a decrease in AChE activity at different stages of adaptation to hypoxia induced by IHT (days 5, 10, and 14). Thus, the IHT method significantly mobilises the reserve capacity of oxygen-dependent processes through the system of CA, ACh-AChE mediated by nitric oxide.

## 1. Introduction

The advancement of civilisation and sedentary lifestyles contribute to the prevalence of chronic non-communicable diseases in society, leading to organ dysfunction and physiological imbalances [1]. Research consistently shows that physical activity, particularly in the form of aerobic exercise training and hypoxic training, serves as a powerful preventive measure against chronic metabolic diseases [2]. Physical activity performed in hypoxic conditions significantly increases the effectiveness of both preventive and therapeutic interventions [3].

The physiological properties of the autonomic nervous system, whose two main components are the sympathetic nervous system and the parasympathetic nervous system, have opposing functions in the regulation of many physiological processes that are altered in stress conditions [4,5,6,7,8,9,10,11,12]. The brain and internal organs have been shown to communicate extensively through the autonomic nervous system. The vagus nerve plays a critical role in this communication [13]. Recent studies have highlighted the role of the vagus nerve in modulating inflammation through multiple pathways [13]. These include the hypothalamic-pituitary-adrenal axis, which releases cortisol, and the cholinergic anti-inflammatory pathway. The latter involves the release of acetylcholine (ACh) at synapses with macrophages. It is important to note that acetylcholine is not only a neurotransmitter. It is also a free mediator that can affect immune cells, such as macrophages and lymphocytes. The properties of acetylcholine in inflammation have been demonstrated in a number of studies [14,15]. Binding acetylcholine to muscarinic [16] and nicotinic receptors [17] on these cells leads to reduced production of pro-inflammatory cytokines (e.g., TNF-α, IL-1β, IL-6), resulting in a lower overall inflammatory state in the body [18]. Equally important is the ability to reduce oxidative stress, as inflammatory processes often increase the production of reactive oxygen species (ROS), which can damage cells and tissues. Through its anti-inflammatory effects, acetylcholine helps to reduce the production of ROS [19]. By reducing oxidative damage, acetylcholine promotes tissue health and regeneration.

**Figure 1:**
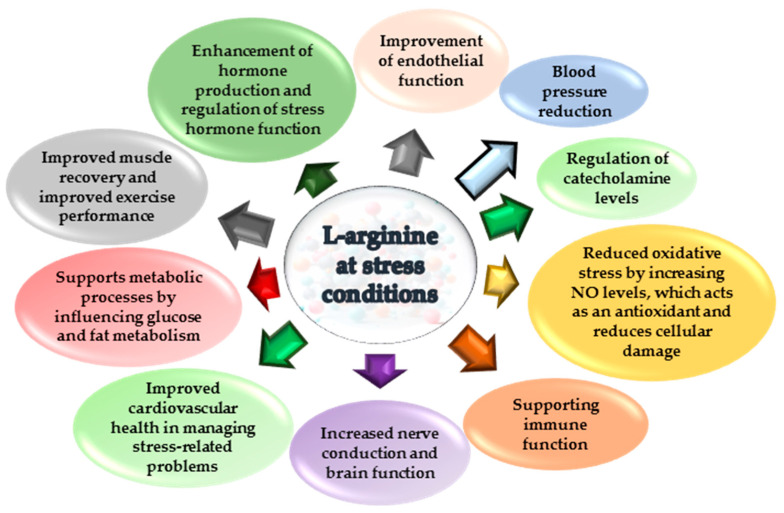
Effects of the amino acid L-arginine during stress.

Adaptation to intermittent hypoxia attenuates the overstimulation of endothelial vasodilator function that often results from excessive nitric oxide production and its increased binding to physiologically active reservoirs such as haem and non-haem iron and thiols [20,21,22,23,24]. Intermittent hypoxia training (IHT), also known as intermittent hypoxia adaptation, involves periodic exposure of the body to low oxygen levels, which leads to a number of physiological adaptations. Furthermore, IHT can improve metabolic efficiency by affecting mitochondrial function, resulting in increased oxidative enzyme activity and mitochondrial oxygen utilisation, improving cellular energy efficiency [20,23,25]. The role of the nitric oxide (NO) system and L-arginine in stress conditions is crucial [26]. Nitric oxide acts as a mediator by dilating blood vessels, thereby improving blood flow and oxygen delivery to tissues under stress [24,27]. As a precursor for NO biosynthesis, L-arginine is essential for NO synthesis and plays a crucial role in mitochondrial oxidation processes [28], and the system of nitric oxide metabolism via nitric oxide synthase functions because NOS synthase converts L-arginine into NO and citrulline, as shown as a pharmacological effect [29]. In stress conditions, the availability of L-arginine influences NO levels and, consequently, the body’s ability to regulate vascular responses and blood pressure. In particular, NO has antioxidant properties and helps to reduce oxidative stress associated with physiological and psychological stress [30], as shown in Figure 1.

The study highlights the potential therapeutic applications of IHT in diseases requiring improved oxygen delivery and metabolic efficiency. Another of our investigations [31] focused on the effects of IHT on mitochondrial oxygen consumption in rats subjected to skeletal unloading. This study showed that IHT resulted in significant changes in mitochondrial function, demonstrating increased mitochondrial oxygen consumption and improved oxidative phosphorylation efficiency. Our team has previously studied the interplay between Krebs cycle intermediates and the nitric oxide system under hypoxia and uncovered the complex mutual and competitive relationships that further unravel the complex mechanisms of IHT effects on cellular and metabolic processes [27].

The studies presented in this paper are a continuation of this work, elucidating the role of oxygen-dependent processes initiated by acute stress after animals have a potential reserve of adaptive metabolic capacity initiated by sessions of IHT. Therefore, the aim of this study was to investigate how the sympathetic nervous system, through the action of adrenaline, noradrenaline, dopamine, and DOPA, is activated in stress conditions, leading to a series of adaptive responses. We also wanted to examine the role of the parasympathetic nervous system, in particular the acetylcholine-cholinesterase system, in this context. Through its primary biosynthetic precursor L-arginine, the nitric oxide system plays a crucial role in the regulation of stress responses, in particular by effective modulation of oxygen-dependent processes and reduction of oxidative stress [24]. The integration of these processes is essential for effective stress management and maintenance of organismal homeostasis. Therefore, the protective effects of adaptation to various environmental factors can be reproduced with NO donors [32]. It has been established that the stress response is realised by a change (mainly an increase) in the production of mediators and hormones by components of the stress system and related structures of stress-limiting systems of the organism [33,34]. However, the changes in catecholamine (CA) levels in rats during administration of the precursor of nitric oxide biosynthesis, L-arginine, and the inhibitor of NOS synthase, N^ω^-nitro-L-arginine (L-NNA), under the influence of acute stress to IHT-treated rats have not been fully elucidated. The choice of liver mitochondria in this study stems from their central role in regulating the body’s energy metabolism and their particular sensitivity to physiological changes associated with the nervous and vascular systems. Liver mitochondria are responsible for essential metabolic processes such as gluconeogenesis and ATP synthesis, which can be directly influenced by neurohormonal signals. By studying liver mitochondria, we aim to better understand how changes in the nervous and vascular systems affect cellular metabolism. In addition, the liver serves as a central organ integrating different metabolic signals, making liver mitochondria an ideal model for analysing the influence of these systems on energy functions. This approach allows us to gain valuable insights into the physiological interactions between the nervous, vascular and metabolic systems.

The aims of this study were: (1) to assess the combined effects of acute emotional stress and intermittent hypoxia on oxygen-dependent processes in rats, focusing on mitochondrial oxidative phosphorylation, microsomal oxidation, and the intensity of lipoperoxidation processes, (2) to evaluate how the modulating effect of the NO synthase mechanism via L-arginine and L-NNA on the concentration of catecholamines and their biosynthetic precursors varies depending on the intermittent hypoxia-generating processes in rats, (3) to investigate the molecular mechanisms underlying the protective stress-limiting effects of intermittent hypoxic training on energy metabolism using oxygen-dependent processes, in particular the dependence of microsomal oxidation and lipid peroxidation, and (4) to analyse the role of acetylcholine (ACh) and acetylcholinesterase (AChE) in the regulatory processes of intermittent hypoxia, with particular reference to their effects on oxygen-dependent processes and the balance between ACh and AChE in the context of adaptation to both stress and hypoxic conditions induced by the interval mode.

## 2. Results

### 2.1. Oxygen-Dependent Processes, Nitric Oxide System, and Stress

In our study, we analysed the role of oxygen-dependent processes of mitochondrial energy supply in the liver using the following Krebs cycle substrates, succinate and alpha-ketoglutarate, and elucidated the role of the first and second complexes of the mitochondrial respiratory chain using corresponding inhibitors. These data are presented in Table 1 and Table 2. In stress conditions, the data on the oxidation of succinate (SC) and α-ketoglutarate (KGL) revealed the preferential oxidation of SC, compared to KGL. This was evidenced by the fact that the rate of phosphorylating respiration in the case of the SC oxidation was statistically higher than the conjugation of respiration and phosphorylation processes determined by the value of the respiratory control ratio according to Chance [35,36,37]. It should be noted that we obtained an increase in the respiratory control ratio at both substrates of oxidation under stress, with a significant decrease in the ADP/O ratio at KGL oxidation. Thus, the stress-induced preferential activation of the oxidative metabolism of the second site of the mitochondrial respiratory chain takes place with a significant attenuation of the role of NADH-related oxidation.

The pre-stress administration of L-arginine to the animals significantly reduced the rate of state 3 phosphorylated respiration and the level of the respiratory control ratio defined by Chance but increased the level of ADP/O during the SC oxidation in the liver mitochondria, compared to the values obtained in the stress-exposed rats. The pre-stress administration of the nitric oxide precursor, the amino acid L-arginine, reduced the state 3 respiratory rate, the level of respiratory control, and the ADP/O ratio during the KGL oxidation in the liver mitochondria, compared to the values obtained in the stress-exposed rats. The pre-stress administration of the nitric oxide synthase inhibitor, N^ω^-nitro-L-arginine (L-NNA), to the rats caused a decrease in the coupling between respiration and phosphorylation during the SC oxidation, without affecting the other parameters, compared to the values obtained in the stress-exposed rats. The administration of L-NNA to the animals before the stress model resulted in significant activation of mitochondrial respiration during the KGL oxidation, but these changes were accompanied by a significant decrease in the coupling between respiration and phosphorylation, as estimated by the respiratory control ratio proposed by Chance (Table 1).

The next stage of our research was devoted to inhibition analysis, which was used to assess the role of different sites in the mitochondrial chain in energy supply processes (Table 2). The contribution of the “net” oxidation of NAD-dependent substrates, as exemplified by the oxidation of KGL, can be significantly modulated under the influence of malonate as a succinate dehydrogenase (SDH) inhibitor, which significantly alters the rates of mitochondrial respiration. This dependence is related to the significant rate of SC oxidation that can alter the oxidation of NAD-dependent substrates. The injection of L-arginine prior to the stress model resulted in a statistically significant increase in mitochondrial oxygen uptake processes (70.6%, *p* < 0.05), with a concomitant increase in the respiratory control ratio described by Chance (37.2%, *p* < 0.05), compared with the data obtained in the stress-exposed rats. Thus, endogenous succinate, whose oxidation rate is maintained by the activity of SDH, can significantly influence the oxidation of KGL during stress. NO-dependent mechanisms play an important role in these processes (Table 2).

The role of NAD-dependent oxidation in SC oxidation using rotenone, an inhibitor of the Complex I of the mitochondrial respiratory chain, is shown in Table 2. The analysis of the effects of rotenone on SC oxidation showed an increase in the rate of state 3 mitochondrial respiration and a decrease in the ADP/O ratio, compared with the untreated controls. The effects of the amino acid L-arginine were associated with a marked decrease in mitochondrial respiratory processes and their efficiency, with a concomitant increase in the respiratory control ratio defined by Chance [35,36,37], compared with the data obtained in the stress-exposed rats. The administration of the nitric oxide synthase inhibitor L-NNA to the rats prior to the stress exposure reduced mitochondrial respiratory processes statistically significantly but was accompanied by high levels of phosphorylation efficiency ADP/O ratio), compared to the data obtained in the stress-exposed rats. Thus, the effect of the precursor of nitric oxide synthesis was associated with the maintenance of NAD-dependent oxidation in mitochondria in the conditions of simulated acute emotional stress in the rats, which is more damaging in stress conditions, compared to SC oxidation. As will be shown below, this is probably one of the causes of the depletion of respiratory activity reserves in stress conditions under the influence of significant concentrations of catecholamines (Table 2).

The various possibilities considered for the progressive inhibition of succinate dehydrogenase in stress conditions can be related to the influence of factors with varying intensity. Glutamate is thought to play an important role in these processes. We therefore investigated the changes in mitochondrial respiratory processes in stress conditions and their modulation by the NO system using a mixture of substrates, such as succinate and glutamate, in the presence of the inhibitor rotenone (Table 2). It was shown that the oxidation of SC, under inhibition of NAD-dependent oxidation and removal of the progressive inhibition of the enzyme SDH, leads to the activation of phosphorylation respiration, which, however, is not coupled to oxidative phosphorylation and occurs with lower efficiency (we observed a decrease in the respiratory control ratio defined by Chance and the ADP/O ratio, *p* < 0.05), compared to the data obtained in the untreated control rats. The effects of increasing the role of the NO system during the administration of L-arginine to the stress-exposed rats were revealed by the decreased values of the rate of mitochondrial phosphorylation but the increased efficiency and coupling between respiration and oxidative phosphorylation. The effects of the nitric oxide synthase inhibitor L-NNA in these conditions were accompanied by the activation of the rate of state 3 mitochondrial respiration as well as its coupling and efficiency, compared to the stress conditions, but these changes were significantly lower, compared to the effects of L-arginine. Thus, the maintenance of succinate-dependent oxidation and the functional role of NAD-dependent oxidation of substrates in the mitochondrial respiratory chain under stress are mutually dependent on the role of the nitric oxide system.

### 2.2. Catecholamines and Nitric Oxide System During Stress

**Figure 2:**
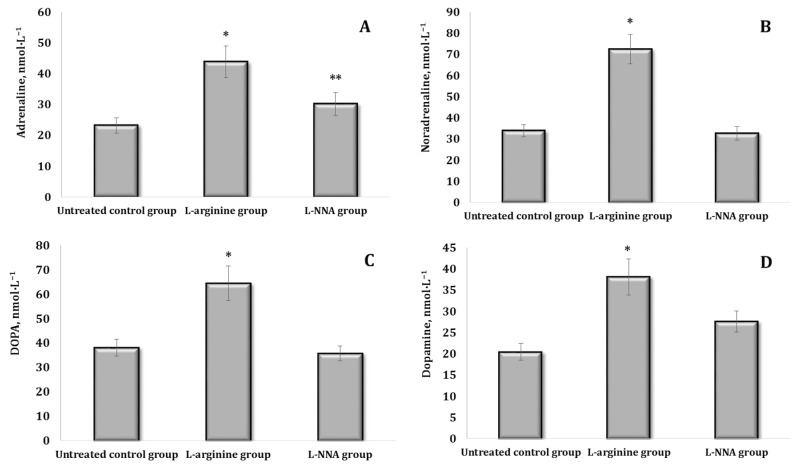
Content of catecholamines [adrenaline, AD (**A**), noradrenaline, NA (**B**), DOPA (**C**), and dopamine, DA (**D**)] in the blood (nmol∙L^−1^) of rats treated with L-arginine (600 mg/kg, 30 min) or L-NNA (35 mg/kg, 30 min). Data expressed as mean ± SD (*n* = 6). * Significant differences between the untreated control group and the L-arginine group (*p* < 0.05), ** Significant differences between the untreated control group and the L-NNA group (*p* < 0.05).

Since the stress reaction is implemented by a change (mainly an increase) in the production of mediators and hormones by components of the stress system and related structures of the stress-limiting systems of the organism, we analysed changes in the content of catecholamines (CA) and their precursors in the blood of rats upon the parenteral administration of L-arginine or the inhibitor of nitric oxide synthase L-NNA. These data are presented in Figure 2.

It was shown that the effect of L-arginine after 30 min was accompanied by a probable increase in all the above-mentioned indices in the blood of rats, but the percentage of the increase varied. The levels of adrenaline (AD), noradrenaline (NA), and DOPA almost doubled in these conditions, and dopamine (DA) increased by 77.5% (*p* < 0.05) (Figure 2A–D). Under the influence of L-NNA, the AD content increased by 55.2% (*p* < 0.05), (Figure 2A). Thus, under the influence of L-arginine, there was an increase in the levels of all CAs and their precursors, especially NA. The nitric oxide synthase inhibitor L-NNA leads to a preferential increase in AD but not in its biosynthetic precursors NA and DOPA (Figure 2B–D).

**Figure 3:**
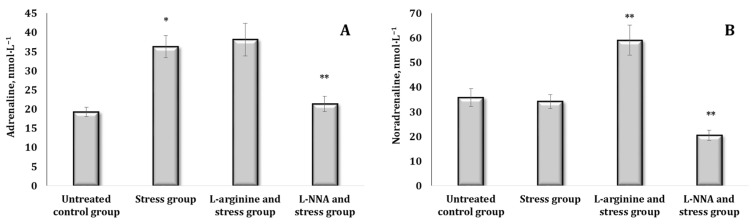

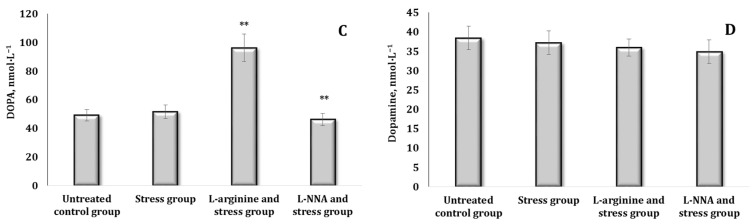
Content of catecholamines [adrenaline, AD (**A**), noradrenaline, NA (**B**), DOPA (**C**), and dopamine, DA (**D**)] in the blood (nmol∙L^−1^) of rats exposed to stress conditions and after administration of L-arginine (600 mg/kg, 30 min) or L-NNA (35 mg/kg, 30 min) in stress-induced conditions. Data expressed as mean ± SD (*n* = 6).* Significant differences between the stress group and the untreated control group (*p* < 0.05), ** Significant differences between the stress group and L-arginine + stress or the L-NNA + stress groups (*p* < 0.05).

The method of stressing the animals used in the current study (“free floating in a cage”) indicated disturbances in the system of CA biosynthesis accompanied by an increase in the content of primarily AD (by 54%, *p* < 0.05). These changes in catecholamines in the blood of the animals are shown in Figure 3. No reliable changes in the indices were observed in these conditions for the other CAs (other than the indicated AD) and their biosynthetic precursors (Figure 3A). The exposure to L-arginine in stress conditions was likely to increase the levels of all the indicators studied, with the exception of DA levels (Figure 3B,C).

**Figure 4:**
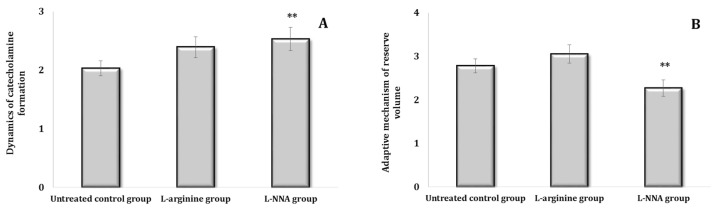
Dynamics of catecholamine formation (DCF, (**A**)) and the adaptive mechanism of reserve volume (AMRV, (**B**)) in rats treated with L-arginine (600 mg/kg, 30 min) or L-NNA (35 mg/kg, 30 min). Data expressed as mean ± SD (*n* = 6). ** Significant differences between the untreated control group and the L-NNA group (*p* < 0.05).

The state of some links of the sympathoadrenal system can be clarified by the ratio of NA/AD in the blood, which increases with the strengthening of the mediator link and decreases with the hormonal increase. For a relative assessment of the processes of the dynamics of CA formation, the ratio of the sum of AD and NA to DA is calculated. The ratio of the sum of AD, NA, and DA to DOPA, which indicates the accumulation of DOPA and increased transfer to dopamine in NA, is regarded as the size of the reserve volume of adaptation mechanisms. The ratio of the first to the second coefficient is an indicator of changes in the reserve capacity of the sympathoadrenal system. The dynamics of CA formation calculated with this method for experimental data indicated an increase in these capacities during in single parenteral administration of L-arginine and their decrease in the L-NNA action conditions. The stress test (analysis of the data in Figure 4A,B) also showed an increase in the reserve volume of the adaptation mechanisms in comparison with the control values.

**Figure 5:**
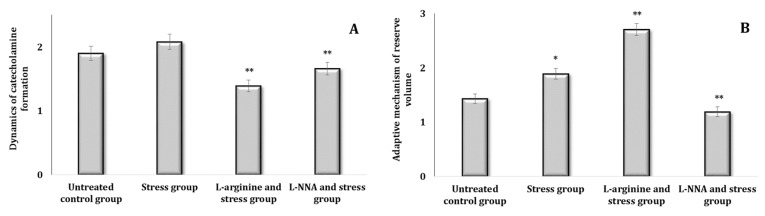
Dynamics of catecholamine formation (DCF, (**A**)) and the adaptive mechanism of reserve volume (AMRV, (**B**)) in rats treated with L-arginine (600 mg/kg, 30 min) or L-NNA (35 mg/kg, 30 min) before exposure to stress. Data expressed as mean ± SD (*n* = 6). * Significant differences between the stress group and the untreated control group (*p* < 0.05), ** Significant differences between the stress group and the L-arginine + stress or the L-NNA + stress groups (*p* < 0.05).

The parenteral administration of L-arginine 30 min before the stress model was associated with an increase in the compensatory capacity of the organism in conditions of extreme stress: the first coefficient decreased and the second increased. The nitric oxide synthase inhibitor L-NNA administered before the stress model did not cause an increase in this ratio of coefficients, because it led to opposite changes caused by the administration of L-arginine (analysis of the data in Figure 5A,B). The administration of the precursor of NO biosynthesis, L-arginine, was therefore accompanied by an increase in the compensatory capacity of the sympathoadrenal system under stress, which can be used to correct the undesirable effects of stress.

Thus, the parenteral administration of L-arginine prior to exposure to extreme stress shows a marked improvement in the body’s compensatory capacity. This effect is evidenced by a decrease in the NA/AD ratio, indicating a favourable modulation of the sympathetic response, which strengthens the mediator link within the sympathoadrenal system. In addition, the observed increase in the second coefficient suggests improved catecholamine synthesis and general adaptive mechanisms. These findings suggest that L-arginine not only facilitates the balance between norepinephrine and adrenaline levels, but also increases the availability of precursors necessary for catecholamine production. Consequently, L-arginine may play a crucial role in optimising the functionality of the sympathoadrenal system, thereby increasing the body’s resilience and ability to cope with extreme stress. This research supports the potential therapeutic use of L-arginine in stress management and highlights its importance in physiological adaptation processes.

### 2.3. Catecholamine-Dependent Component of IHT Formation

**Figure 6:**
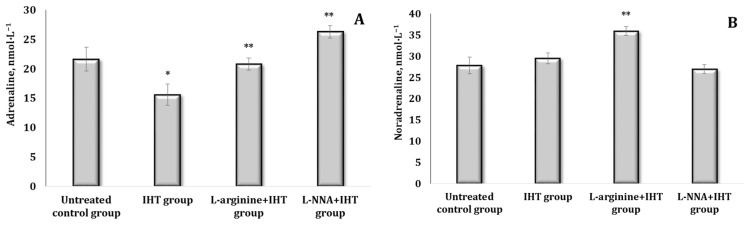

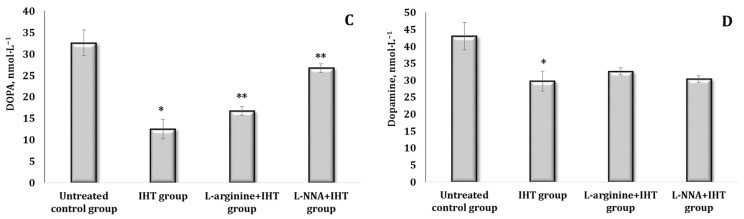
Content of catecholamines [adrenaline, AD (**A**), noradrenaline, NA (**B**), DOPA (**C**), and dopamine, DA (**D**)] in the blood (nmol∙L^−1^) of rats during adaptation to hypoxia in the interval mode (IHT) for 5 days with the administration of L-arginine (600 mg/kg, 30 min) or L-NNA (35 mg/kg, 30 min) in stress-induced conditions. Data expressed as mean ± SD (*n* = 6). * Significant differences between the IHT group and the untreated control group (*p* < 0.05), ** Significant differences between the IHT group and the IHT + L-arginine or the IHT + L-NNA groups (*p* < 0.05).

Adaptation to different types of stress is accompanied by changes in CA concentrations, the intensity of which is associated with changes in metabolic regulation. Therefore, our task was to assess the role of CA in the development of the effects of the 14-day IHT. It was shown that a 5-day course of IHT led to a decrease in blood levels of AD, DA, and DOPA in the rats (Figure 6A,C,D). The administration of L-arginine prior to each IHT session enhanced the role of both hormonal and mediator links of the sympathoadrenal system. Both AD and DA levels were increased in these conditions (5th day of IHT, Figure 6A,C). The administration of the NO synthase inhibitor L-NNA was accompanied by an increase in AD and DOPA on the 5th day of adaptation, without changes in the levels of NA and DA in the blood of the rats (Figure 6A,C).

**Figure 7:**
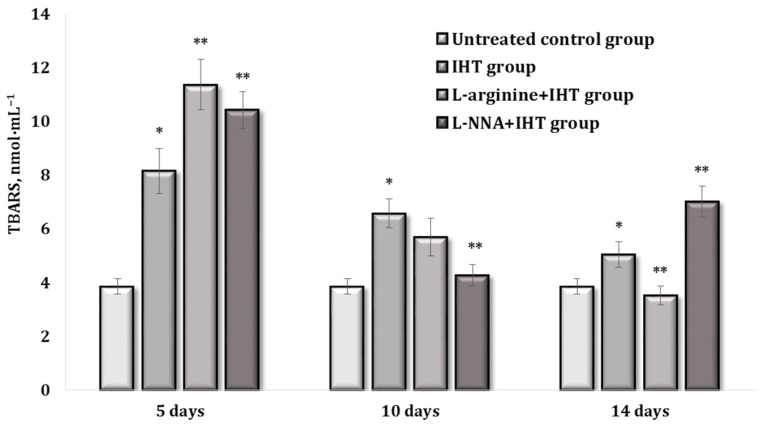
TBARS content (nmol∙mL^−1^) in the blood of rats during adaptation to hypoxia in the interval mode (IHT) on the 5th, 10th, and 14th day of the IHT impact and the administration of L-arginine (600 mg/kg, 30 min) or the nitric oxide synthase inhibitor L-NNA (35 mg/kg, 30 min). Data expressed as mean ± SD (*n* = 6). * Significant differences between the IHT group and the untreated control group (*p* < 0.05), ** Significant differences between the IHT group and the IHT + L-arginine or the IHT + L-NNA groups (*p* < 0.05).

Simultaneously with the formation of adaptation mechanisms to interval hypoxia and IHT with modulation of the activity of the NO system in relation to changes in the level of CA, we observed statistically significant changes associated with the intensification of lipoperoxidation processes, assessed by the level of TBARS, in these three periods of the study (on the 5th, 10th, and 14th days of the study), which is shown in Figure 7. On the fifth day of the study, we obtained a statistically significant increase of 112% (*p* < 0.01) in the intensity of lipoperoxidation processes in the blood, compared with the data obtained in the control group. It was still increased (by 39.5%, *p* < 0.05) when the amino acid L-arginine and the nitric oxide synthase inhibitor L-NNA were administered (by 27.9%, *p* < 0.05) in relation to the data obtained in the IHT-exposed group. Thus, the formation of IHT in the early stages of the process was accompanied by a decrease in the AD concentration, and these dependencies were correctable by the pharmacological agents of the nitric oxide system. However, an important detail of the initial stage of IHT formation is associated with the intensification of lipoperoxidation processes in which metabolic signalling pathways play an important role.

**Figure 8:**
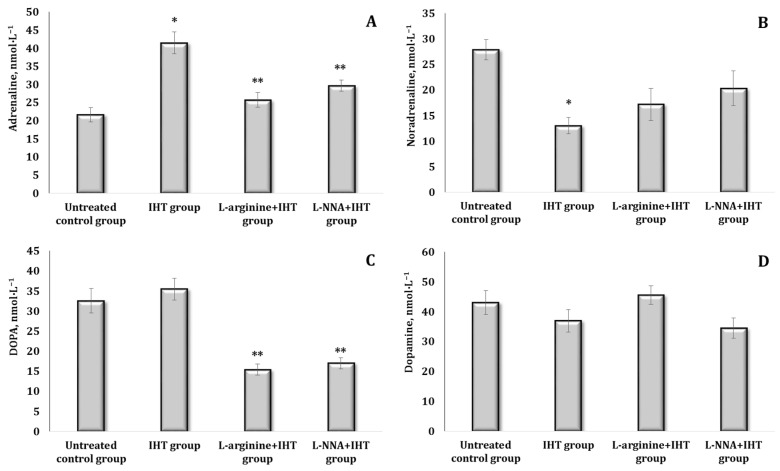
Content of catecholamines [adrenaline, AD (**A**), noradrenaline, NA (**B**), DOPA (**C**), and dopamine, DA (**D**)] in the blood (nmol∙L^−1^) of rats during adaptation to hypoxia in the interval mode (IHT) for 10 days and the administration of L-arginine (600 mg/kg, 30 min) or L-NNA (35 mg/kg, 30 min) in stress-induced conditions. Data expressed as mean ± SD (*n* = 6). * Significant differences between the IHT group and the untreated control group (*p* < 0.05), ** Significant differences between the IHT group and the IHT + L-arginine or the IHT + L-NNA groups (*p* < 0.05).

The course of adaptation to hypoxia in the interval mode was accompanied by an increased blood concentration of expressed AD and a decreased NA on day 10 of the experiment (Figure 8A,B). The administration of both L-arginine and the nitric oxide synthase inhibitor L-NNA caused a statistically significant decrease in both AD and DOPA, compared with the data obtained in the IHT-exposed group. We also observed that the L-NNA administration increased the NA levels, compared to IHT, and the L-arginine administration caused an increase in DA, compared to the data obtained in the IHT-exposed group. It is interesting to note that the content of TBARS on the 10th day of adaptation was 70.7% (*p* < 0.05) higher in relation to the data obtained in the control group, but was reduced by 24% (*p* < 0.05) in relation to the initial stage of IHT (on the 5th day), as shown in Figure 6. Thus, the formation of IHT at the initial stages of the process is accompanied by modulation of the catecholamine concentration, these dependencies of IHT formation are related to the reduction of the intensification of lipoperoxidation processes, which are linked.

**Figure 9:**
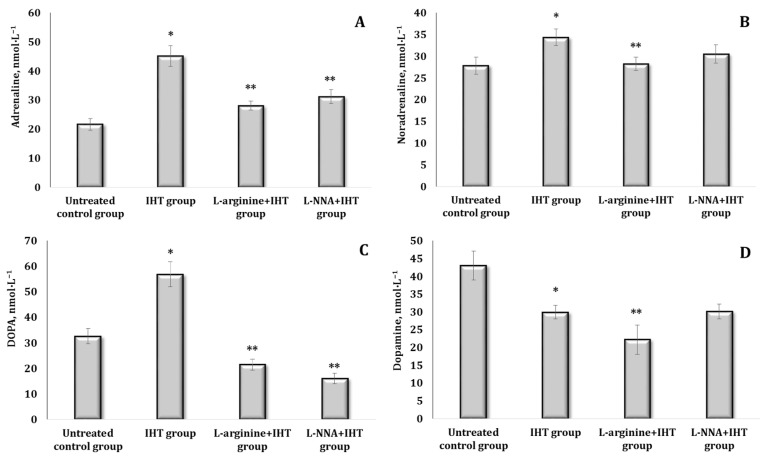
Content of catecholamines [adrenaline, AD (**A**), noradrenaline, NA (**B**), DOPA (**C**), and dopamine, DA (**D**)] in the blood (nmol∙L^−1^) of rats during adaptation to hypoxia in the interval mode (IHT) for 14 days and the administration of L-arginine (600 mg/kg, 30 min) or L-NNA (35 mg/kg, 30 min) in stress-induced conditions. Data expressed as mean ± SD (*n* = 6). * Significant differences between the IHT group and the untreated control group (*p* < 0.05), ** Significant differences between the IHT group and the IHT + L-arginine group or the IHT + L-NNA groups (*p* < 0.05).

The course of adaptation to hypoxia in the interval mode was accompanied by increased blood concentrations of AD, NA, and DOPA and decreased DA on day 14 of the experiment (Figure 9A–D). The administration of L-arginine and the nitric oxide synthase inhibitor L-NNA caused a statistically significant decrease in both AD and DOPA, compared to the data obtained during IHT (Figure 9A–D). We also observed a decrease in DA when L-arginine was administered, compared to the data obtained by IHT. The content of TBARS products was only 30.1% (*p* < 0.05) higher on the 14th day of adaptation, compared to the data obtained in the control group, but was reduced by 7.7%, compared to the initial stage (10th day) of IHT (Figure 8). The introduction of L-arginine was associated with a further reduction in blood TBARS levels by 69% (*p* < 0.01), compared with the data obtained in the IHT-exposed group on day 5 and 38.1% (*p* < 0.05) on day 10 of IHT exposure, compared with the data obtained in the IHT-exposed group on day 14. The administration of L-NNA on day 14 of the IHT exposure was associated with mitigation of the effects of L-arginine, as the value was higher than both the data obtained in the IHT-exposed group on day 14 (by 39%, *p* < 0.05) and the data obtained in the L-arginine administration group (by 99.2%, *p* < 0.01) as shown ((Figure 9A,C).

Thus, the IHT exposure in the final stages of this process is accompanied by an increase in the concentration of CA, and these dependencies of IHT formation are associated with a pronounced decrease in the intensification of lipoperoxidation processes, which are enhanced by the injection of the precursor of nitric oxide biosynthesis, i.e., the amino acid L-arginine.

### 2.4. Acetylcholine, Acetylcholinesterase, and IHT Exposure

**Figure 10:**
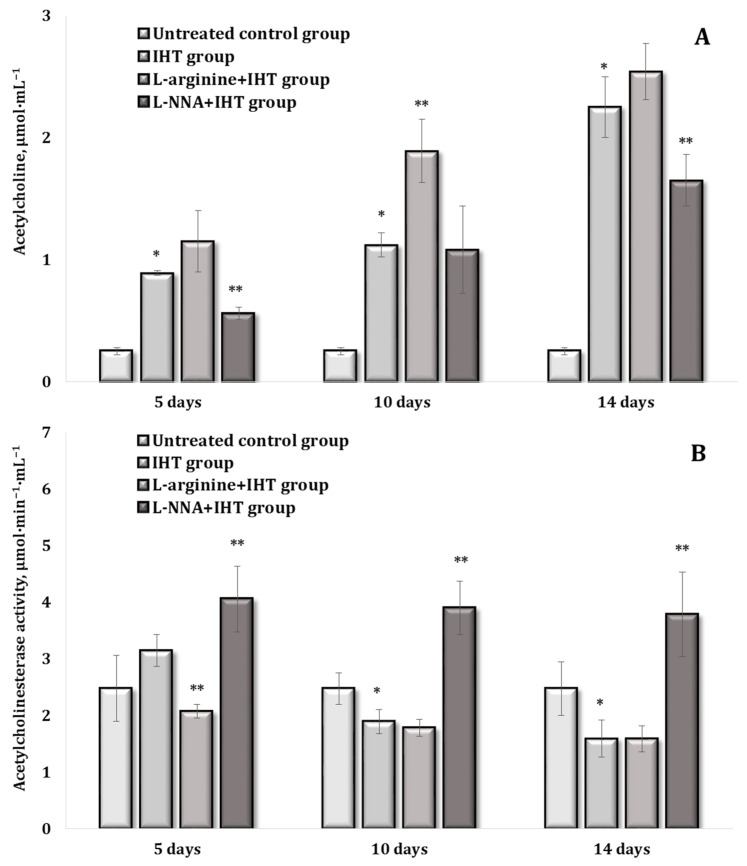
Acetylcholine content ((**A**), µmol∙mL^−1^) and acetylcholine esterase activity ((**B**), µmol∙min^−1^∙mL^−1^) in the blood of rats during adaptation to hypoxia in the interval mode (IHT) on the 5th, 10th, and 14th day of the IHT impact and the administration of L-arginine (600 mg/kg, 30 min) or the nitric oxide synthase inhibitor L-NNA (35 mg/kg, 30 min). Data expressed as mean ± SD (*n* = 6). * Significant differences between the IHT group and the untreated control group (*p* < 0.05), ** Significant differences between the IHT group and the IHT + L-arginine or the IHT + L-NNA groups (*p* < 0.05).

Acetylcholine (ACh), which acts as both a neurotransmitter and a signalling molecule, plays a crucial role in regulating inflammatory responses and oxidative stress and promotes tissue regeneration after hypoxia, its ability to modulate inflammatory processes and support tissue repair is essential for maintaining homeostasis and health, especially in hypoxic stress conditions, making it a key factor in the body’s adaptation to challenging environmental conditions and in supporting regenerative processes (Figure 10A). In addition, the enzyme acetylcholinesterase (AChE) is also important in this context, which is why we investigated this system on days 5, 10, and 14 of the IHT exposure, as shown in Figure 10B. In our studies, we observed a statistically significant increase in the levels of both catecholamines and acetylcholine in the blood against a background of a decrease in the activity of its hydrolysing enzyme acetylcholinesterase (AChE) both at the beginning (day 5), in the middle (day 10), and at the end (day 14) of the adaptation to hypoxia. IHT was shown to significantly mobilise the reserve capacity of oxygen-dependent processes via the ACh-AChE system.

This was achieved by correcting metabolic reactivity involving the acetylcholine-acetylcholinesterase system. Importantly, these mechanisms were enhanced by the administration of the amino acid L-arginine and attenuated by the administration of the nitric oxide synthase inhibitor L-NNA during IHT, compared to the IHT alone treatment. Therefore, through the action of the amino acid L-arginine during the IHT exposure, the nitric oxide system enhanced the metabolic benefits of the processes of mitochondrial function by reducing oxidative stress and conserving the use of oxygen in these processes.

### 2.5. IHT, Nitric Oxide System, and Stress

Our studies showed modulating effects of the mutual influence of these factors in relation to the oxidation of succinate and ketoglutarate substrates in mitochondria in animals that had undergone a course of adaptation to hypoxia in the interval mode, compared to the data obtained in the control group (Table 3). These changes were associated with an increase in the coupling of respiratory and oxidative phosphorylation processes in the mitochondria, as assessed by the level of the respiratory control ratio proposed by Chance as well as the phosphorylation efficiency (ADP/O ratio) in the SC oxidation, compared to the data obtained in the control group. The effects of adaptation to hypoxia by IHT exposure in stress conditions were rather due to the enhanced oxidative effects of KGL, compared to SC, namely relative to the rate of oxidative phosphorylation in state 3, the respiratory control ratio defined by Chance [34,35,36], and the phosphorylation efficiency (ADP/O ratio). The administration of L-arginine in these conditions was associated with the maintenance of the effects of the preferential oxidation of KGL over SC and was significantly modified by the nitric oxide synthase inhibitor during the stress exposure of the IHT-treated rats.

As shown in our study, the activity of the cytochrome P_450_-dependent enzyme aminopyrine N-demethylase was significantly altered during the stress exposure in the IHT-treated rats. Importantly, this enzyme plays a critical role in the metabolism of aminopyrine, a compound often used as an experimental model substrate to assess the activity of cytochrome P_450_ enzymes. It was shown that the activity of cytochrome P_450_ enzymes, including aminopyrine N-demethylase, was altered in the stress and hypoxic conditions (decreased by 25.78%, *p* < 0.05), affecting the oxygen-dependent type of microsomal oxidation processes in metabolism. The administration of L-arginine resulted in a decrease in aminopyrine N-demethylase activity (by 37.45%, *p* < 0.01), compared with the data obtained during the stress exposure in the IHT-treated rats. Understanding the role of aminopyrine N-demethylase activity is important, as it contributes to the metabolic adaptation of organisms in challenging environmental conditions and provides insights into effective levels of microsomal oxidation and physiological responses in stress-related conditions. These processes involved a significant increase in lipid peroxidation in the blood and liver of rats during the IHT and stress exposure. Similar effects of IHT and stress were observed after the L-arginine administration. The effects were mitigated by the L-NNA administration in the IHT-treated stress-exposed rats, compared to the data obtained in the IHT-treated rats in stress conditions.

**Figure 11:**
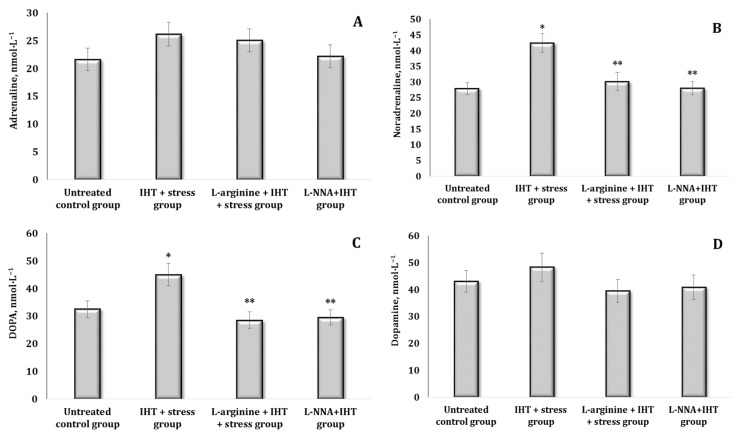
Content of catecholamines [adrenaline, AD (**A**), noradrenaline, NA (**B**), DOPA (**C**), and dopamine, DA (**D**)] in the blood (nmol∙L^−1^) of IHT-treated rats exposed to stress and treated with L-arginine (600 mg/kg, 30 min) or the nitric oxide synthase inhibitor L-NNA (35 mg/kg, 30 min) and exposed to stress. Data expressed as mean ± SD (*n* = 6). * Significant differences between the IHT + stress group and the untreated control group (*p* < 0.05), ** Significant differences between the IHT + stress group and the L-arginine + IHT + stress or the L-NNA + IHT + stress groups (*p* < 0.05).

**Figure 12:**
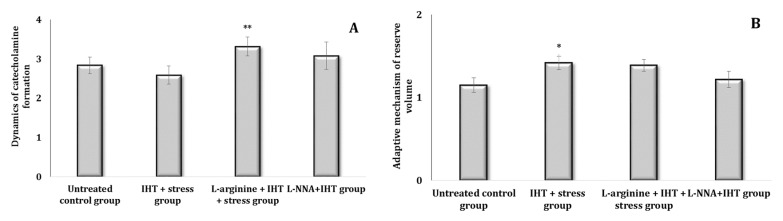
Dynamics of catecholamine formation (DCF, (**A**)) and the adaptive mechanism of reserve volume (AMRV, (**B**)) in IHT-treated rats exposed to stress and treated with L-arginine (600 mg/kg, 30 min) or the nitric oxide synthase inhibitor L-NNA (35 mg/kg, 30 min) and exposed to stress. Data expressed as mean ± SD (*n* = 6). * Significant differences between the IHT + stress group and the untreated control group (*p* < 0.05), ** Significant differences between the IHT + stress group and the L-arginine + IHT + stress group or the L-NNA + IHT + stress groups (*p* < 0.05).

The influence of stress in the IHT-treated animals was accompanied by modulation of the biochemical effects on the levels of catecholamines and precursors of their biosynthesis, as shown in Figure 10. This was manifested by the absence of changes in blood levels of AD, as in the IHT-treated rats exposed to the stress model, when L-arginine and L-NNA were administered. However, statistically significant increases in NA and DOPA levels were produced by the administration of L-arginine to the IHT-treated rats followed by the stress exposure. These features of changes in the functional state of the catecholamine system were manifested in the parameters of the dynamics of catecholamine formation (DCF, A) and the reserve volume of the adaptive mechanism (AMRV, B) in the blood of the rats, which are shown in Figure 11. Importantly, understanding DCF and AMRV provides insight into how organisms form adaptations to IHT and stress. These parameters show how the organism dynamically adjusts its physiological responses to optimise the effects of stress. The injection of L-arginine into the IHT-treated rats exposed to stress was accompanied by an increase in the dynamics of catecholamine formation as well as the reserve capacity of the sympathoadrenal system (Figure 12A,B).

In conclusion, our studies have highlighted the interplay between different factors influencing substrate oxidation in the mitochondria of IHT-treated animals exposed to stress. The injection of L-arginine into the IHT-treated rats maintained the preferential oxidation of KGL over SC. These effects were reversed by the administration of a nitric oxide synthase inhibitor, L-NNA, in stress conditions. Furthermore, modulated levels of catecholamines and their biosynthetic precursors were observed in the IHT-treated rats exposed to the stress model. These changes in the functional state of the CA system, reflected in the dynamics of CA formation and the reserve volume of adaptive mechanisms, indicate how organisms dynamically adjust their physiological responses to optimise stress adaptation. Overall, understanding these dynamics provides critical insights into how organisms adapt to IHT and stress conditions, in particular by enhancing the adaptive capacity of the sympathoadrenal system through interventions, such as L-arginine administration.

## 3. Discussion

In this study, we analysed the effects of L-arginine or L-NNA administration on mitochondrial oxidation and phosphorylation processes, with particular focus on the oxidation of different mitochondrial substrates and using inhibition analysis in the oxidation of FAD and NADH substrates in mitochondria using the rate of mitochondrial oxidation in state 3, respiratory control ratio, and phosphorylation efficiency levels. We also investigated the modulatory effects of CA and its biosynthetic precursors in IHT-treated rats exposed to stress. These aspects provide new insights into the adaptive responses of the sympathoadrenal system and mitochondrial function in stress conditions, providing deeper understanding of physiological adaptations to challenging environmental stimuli, which we highlight below.

Our study highlights the important role of nitrite in interval hypoxia conditions, particularly through its association with nitric oxide (NO). Traditionally, nitrite anions in humans have been regarded as transient intermediates in the oxidation of nitric oxide radicals to stable nitrate, a process considered irreversible in physiological conditions. However, recent data indicate that endogenous nitrite plays a critical role in the regulation of several signalling events along the physiological and pathophysiological oxygen gradient [23,38].

**Figure 13:**
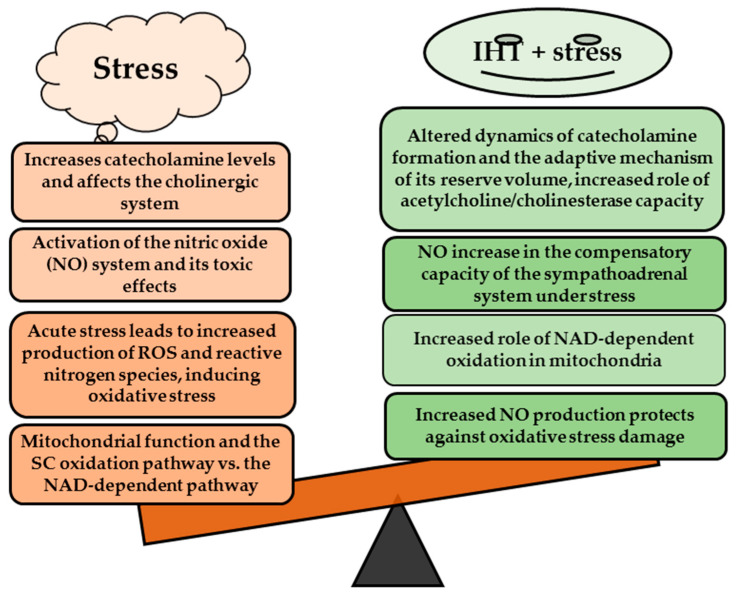
Complexity of the body’s response to acute stress and IHT involving different biochemical and physiological mechanisms.

First, in the stress-exposed rats, the sympathetic nervous system was activated, triggering a number of adaptive responses, while the parasympathetic nervous system was inhibited. IHT via the nitric oxide system, through its primary biosynthetic precursor L-arginine, plays a crucial role in the regulation of stress-activated responses, in particular by modulating mitochondrial respiration levels through SC and KGL oxidation as well as microsomal oxidation and by reducing oxidative stress in the blood and liver of rats. As shown in our study, the integration of these processes is essential for effective stress management and maintenance of organismal homeostasis (Figure 13).

It is known that adaptation to hypoxic conditions has numerous protective effects on the functioning of practically all major systems of the organism, including the cardiovascular, respiratory, nervous, hormonal, and muscular systems [39]. Interval hypoxic training is used in the clinic as a method of increasing the non-specific resistance of the organism, which has a wide range of therapeutic effects on many systems when their functioning is disturbed [40]. The intermittent mode of mixture inhalation in these conditions allows imitating the natural physiological cyclic state of moderate hypoxia caused by auto-oscillations of regulatory systems responsible for tissue respiration, in which the sympathetic and parasympathetic regulatory effects of the autonomic nervous system play an important role [41]. Data on significant changes in NO production and its generating systems confirm the formation of protective effects of IHT and the resulting long-term adaptation [23].

The elucidation of the cellular mechanisms of the protective effect of the IHT method on the processes of energy supply, peripheral blood parameters, tissue metabolism, changes in lipid peroxidation, and the antioxidant defence system of the organism, in combination with the factors of metabolite therapy may form the basis of a comprehensive approach to the protection of tissues from stressor-induced damage [39]. It has been shown that, during adaptation to periodic hypoxia, in organs and tissues there are changes in the expression of genes encoding different forms of NO synthases and vascular NO-dependent responses [42]. This is associated with involvement in limiting cellular damage and the development of apoptosis [43].

Many questions have arisen about the most effective regimens and the best ways for implementation thereof, as different approaches to IHT have been incorporated into sport, fitness, and the medical and military practice. It has been found that mild levels of hypoxia may not provide a sufficient stimulus to activate adaptive mechanisms, while more intense or prolonged levels of hypoxia may lead to detrimental pathological effects. In addition, incorporating relaxation techniques into the lives of modern people, such as meditation, yoga, massage, and sauna sessions into the daily routine, can support IHT, enhance its health benefits, and mitigate any potential risks [44].

In interval hypoxia, the key signalling events, such as vasodilation, modulation of mitochondrial respiration, and cytoprotection after ischaemic injury, are mediated by the reduction of nitrite anions to nitric oxide when local tissue oxygen levels are reduced. This reduction process activates an increasing number of enzymatic and non-enzymatic pathways that enhance the adaptive response to hypoxia. These are precisely the effects of mitochondrial respiration shown in this paper. Previously, we have shown similar results using acute hypoxia as a stress factor [38]. We found that the maintenance of NAD-dependent oxidation in mitochondria during the modelling of acute emotional stress in rats is more efficient when a nitric oxide precursor is added. This highlights the importance of nitric oxide in maintaining mitochondrial function and minimising oxidative stress during both the tested effects of hypoxia and acute emotional stress in rats. In addition, pre-stress supplementation with L-arginine improves the body’s ability to compensate, while the nitric oxide synthase inhibitor L-NNA has the opposite effect, further emphasising the role of nitric oxide in stress adaptation.

The differential affinity of terminal sites for oxygen is relevant to the regulation of oxygen-dependent processes in mitochondrial and microsomal redox chains [45]. We investigated the role of cytochrome P_450_-dependent activity in IHT-treated rats exposed to stress conditions because these enzymes may compete with cytochrome oxidase for oxygen, which may alter the role of the mitochondrial respiratory chain, compared to the microsomal chain. Thus, reducing the role of microsomal oxidation processes, compared to mitochondrial oxidation levels during adaptation to hypoxia, increases resistance to stress damage. This is supported by the results of our correlation analysis between the activity of cytochrome P_450_-dependent aminopyrine N-demethylase and TBARS levels in the liver (r = 0.457, *p* = 0.000). The physiological significance of such changes lies in the release of oxygen for the needs of oxidative phosphorylation, as it depends on the concentration of oxygen as a substrate and the direct involvement of NO in these processes. A direct inhibition of microsomal oxidation processes by endogenous nitric oxide cannot be excluded [23].

Secondly, ACh, which acts as both a neurotransmitter and a signalling molecule, plays a crucial role in regulating inflammatory responses and oxidative stress and promotes tissue regeneration after hypoxia [12] homeostasis and health, especially in hypoxic stress conditions, making it a key factor in the body’s adaptation to challenging environmental conditions and supporting regenerative processes [11]. In addition, the AChE enzyme is also important in this context [46]. Our research confirmed these relationships, with the increases in CA coinciding with the increases in ACh concentrations and decreases in AChE activity. This was confirmed by the results of our correlation analysis between ACh levels and TBARS in the blood (r = −0.512, *p* = 0.000) and liver (r = −0.471, *p* = 0.000) of the IHT-treated rats. The adaptation to IHT significantly influenced the physiological changes, with CA and ACh playing key roles in regulating the response to intermittent hypoxia. CA supports energy mobilisation and improves blood flow, while ACh helps to regulate the heart rate and vasodilation and promotes regeneration [47].

Cardiac parasympathetic fibres and some sympathetic fibres terminate in the intracardiac nerve plexus, where not only parasympathetic postganglionic neuron bodies and axons but also afferent neurons and local circuit neurons have been found [48,49]. Adrenergic neurons immunoreactive to catecholamine metabolising enzymes have also been found in the intracardiac nerve plexus, contrary to the classical view of the autonomic nervous system [50]. Relatively recent studies have shown that the majority of parasympathetic postganglionic neurons in the human intracardiac nerve plexus are also immunoreactive for neuronal NO synthase (nNOS). The distribution of nNOS in the cells of the intracardiac nerve plexus suggests that NO is a co-transmitter of ACh at the terminals of these neurons [50]. Previous data have shown that NO can act in an autocrine manner in such structures, leading to increased release of the major neurotransmitter ACh [51]. However, not all authors agree with this view: some studies have shown that only about 40% of VNSP neurons are stained with antibodies against nNOS [52].

The role of NO synthase and catecholamine regulation in the human intracardiac ganglia has also been elucidated. A number of neurons in the intracardiac plexus have been shown to be immunoreactive to the key enzymes involved in catecholamine synthesis and storage, tyrosine hydroxylase, and vesicular monoamine transporter 2 (VMAT2) [53]. In humans and other primates, the proportion of noradrenergic neurons in the intracardiac plexus may be important [54]. A dual choline-noradrenergic phenotype is therefore present in many neurons. The dual phenotype of neurons in the intracardiac nerve plexus has also been found in other animals (guinea pigs, mice, and rats) [50]. The neurons were also immunoreactive to the enzymes of catecholamine metabolism: aromatic amino acid decarboxylase and dopamine β-hydroxylase. In addition, tyrosine hydroxylase immunoreactivity has been found in mouse and guinea pig SIF cells, but the functional role of some mouse epicardial plexus nerves and bundles is mixed, with most expressing either adrenergic or cholinergic markers [55].

Thirdly, our study has shown that the dynamics of catecholamine formation and the reserve volume of the adaptation mechanism are crucial parameters in the formation of adaptation processes (Figure 6, Figure 8 and Figure 9). Catecholamines, such as epinephrine and norepinephrine, play a central role as neurotransmitters and hormones in the body’s response to stress. Their production dynamics, influenced by various physiological and environmental factors, is integral to the regulation of cardiovascular function, metabolism, and stress response [47]. In adaptive processes, the ability to modulate catecholamine levels ensures effective physiological responses to changing conditions, thereby enhancing resilience and survival. The importance of CAs as regulators of adaptive mechanisms is based on their ability to rapidly and intensely influence metabolic processes, stimulate glycogen and fat catabolism, increase blood glucose levels, promote fatty acid oxidation, increase tissue oxygen consumption, increase cardiac output, and provide blood redistribution for optimal tissue use of energy resources, as shown in a number of studies [56].

These dependencies were also shown for the levels of AD and AChE activity (r = −0.714, *p* = 0.000), DOPA, and TBARS in rats’ blood (r = 0.622, *p* = 0.000). High correlations were found between the data during the formation of the mechanisms of interval adaptation dependencies: at the initial stage (day 5) for AD and TBARS (*p* = 0.722, r = 0.000), the levels of norepinephrine and acetylcholine at day 10 of the IHT course (r = 0.542, *p* = 0.000), and between the dopamine content and cholinesterase activity (r = 0.711, *p* = 0.000) at the final day 14 of the IHT treatment.

It has been shown that the inhibitory factors associated with the oxaloacetate inhibition of SDH are oxidation products of serotonin and AD, which accumulate under a strong and prolonged influence on the organism and inhibit SDH activity [57]. Thus, the rapid metabolic inhibition of mitochondrial SDH by oxaloacetate formed from SC at the hormonal level is supported by oxidation products of biogenic amines by monoamine oxidase. These mechanisms provide multiple levels of SDH inhibition [58]. It is important to note that SC formation and oxidation is not only subject to biogenic amine regulation but also induces catecholamine formation itself as well as monoamine oxidase activation [59]. The above relationships form a substrate and hormone system that functions through a system of flavins that can accept electrons from the above substrates.

Fourthly, it has been shown that the nitric oxide synthase inhibitor L-NNA administered prior to stress produced changes directly opposite to those induced by the administration of L-arginine. Thus, the administration of the precursor of nitric oxide biosynthesis, L-arginine, was accompanied by an increase in the compensatory capacity of the sympathoadrenal system in stress conditions, which can be used to correct the undesirable effects of stress through a diet rich in this amino acid. Therefore, many practical tips regarding dietary recommendations, as well as appropriate physical activity that creates favourable mechanisms for nitric oxide accumulation and vasoconstrictive reactions, become the basis of a healthy lifestyle.

**Figure 14:**
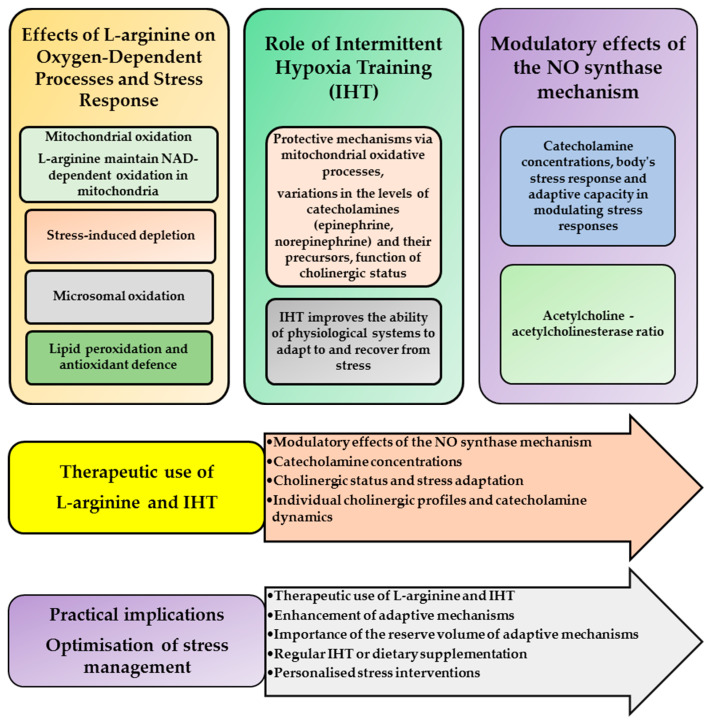
Summary analysis of the effects of L-arginine, intermittent hypoxia training, and acute emotional stress on oxygen-dependent processes and adaptive mechanisms.

In addition, the prospect of clinical studies of NO donors during stress and adaptation is linked to the fact that many diseases and pathological conditions in which stress plays a major role are characterised by a decrease in nitric oxide generating systems [60]. On the other hand, it is known that CAs can form compounds with nitric oxide (6-nitrodopamine and 6-nitronoradrenaline) and modify the state of NO production by acting on nitric oxide synthase [61]). Therefore, the functioning of almost all body systems can be modified by significant catecholamine releases, which are observed during a mismatch in the stimulus-adaptation system as a result of nitric oxide binding in the form of 6-nitrocatecholamines. At the same time, shocks of various origins are characterised by a hyperproduction of nitric oxide, causing a decrease in blood pressure and impaired vasoconstrictor responses, and are associated with the L-arginine paradox described for the corresponding pathological states [62]. As shown in our studies, the exercise stress increased the reserve volume of adaptive mechanisms, compared to the control values, and the introduction of L-arginine into the IHT during exercise modelling was associated with an increase in the compensatory capacity of the organism (Figure 5, Figure 12 and Figure 14).

A number of studies have shown that the central and peripheral links of the stress system have NO-ergic innervation: the neurons of the striatum, midbrain, and hypothalamus contain NO synthase, and the pituitary gland receives extensive NO innervation from the hypothalamus. It has also been shown that NO-ergic neurons innervate the adrenal glands and their axons contact chromaffin cells that produce catecholamines, and sympathetic neurons also contain NA and NO in their terminals, so that the release of NA and NO can occur simultaneously [51,52].

The second important aspect is related to the widely branched system of the cholinergic innervation of the vagus nerve, which has a pronounced immunoprotective effect. This is due to the interrelated effects of nitric oxide and acetylcholine, as nitric oxide can exert its effects via the acetylcholine receptor [63,64]. These authors have shown that acetylcholine induces nitric oxide synthesis in the vascular endothelium, and presumptive in vivo evidence suggests that spinal acetylcholine causes antinociception and increased sympathetic nervous system activity via a nitric oxide mechanism [63].

The reserve volume of adaptive mechanisms refers to the ability of physiological systems to withstand and recover from stressors. This reserve encompasses several mechanisms, including cellular energy production, antioxidant defences, and regulation of stress hormones. It ensures that the body can adapt to environmental challenges and maintain homeostasis despite fluctuations in stress levels. Taken together, the elucidation of the dynamics of catecholamine formation and the reserve volume of adaptive mechanisms provides insights into how organisms form adaptations (Figure 5, Figure 12, Figure 13 and Figure 14). These parameters allow organisms to dynamically adjust their physiological responses to optimise survival in diverse and challenging environments.

### Limitation and Novelty of the Study

In this section, we address the main limitations of our study while highlighting its novel contributions to the field. Understanding these issues is crucial for assessing the impact and scope of our findings as well as their implications for future research.

A limitation of our study is the relatively small sample size, which may affect the generalisability of our findings, a larger sample would increase the robustness and applicability of the results. In addition, our investigation focused on specific stress conditions, potentially excluding other stressors that may influence sympathetic and parasympathetic responses. The methods used to assess mitochondrial respiration, microsomal oxidation, lipid peroxidation, and nitric oxide levels also have inherent limitations, such as variability in biological samples and technical limitations that may affect measurement precision. Finally, the cross-sectional design of our study limits our ability to assess long-term effects and adaptations of the sympathetic and parasympathetic systems over time.

Our study provides a novel and comprehensive analysis of complex oxidative processes, including mitochondrial respiration, microsomal oxidation, and lipid peroxidation, in the context of stress, revealing the intricate regulatory mechanisms and interrelationships between these processes. We also provide new insights into the adaptive mechanisms promoted by the IHT method, highlighting its impact on oxidative processes and stress responses, which enhances our understanding of cellular adaptation and resilience. In addition, our research explores the role of catecholamines (adrenaline, noradrenaline, dopamine, and DOPA) as stress markers and examines their influence on oxidative and adaptive responses. The practical implications of our findings are significant, as they provide valuable insights that could guide the development of targeted therapeutic strategies to manage stress-related disorders and improve cellular health.

Future research should address several key areas that are critical to understanding both the scientific and practical aspects of stress management. Expanding mitochondrial analysis by evaluating additional metabolites and mitochondrial biogenesis will deepen our understanding of cellular energy processes. Investigations of the effects of different types and durations of stress on metabolic functions will help to elucidate stress responses. In addition, further research into cholinergic mechanisms and their influence on catecholamine levels is essential for a complete understanding of stress physiology. Clinical trials evaluating the use of L-arginine and intermittent hypoxia training in humans could provide valuable insights into their practical applications. In addition, testing other dietary supplements and conducting genotyping studies may reveal individual differences in response to interventions, supporting the development of personalised stress management strategies.

## 4. Materials and Methods

### 4.1. Animal Experimental Permission

The experiments were conducted in accordance with the guidelines of the Council of the European Union, the current regulations of Poland and Ukraine, and the recommendations of the Ethics Committee. Approval was granted by the Ethics Committee of T.H. Shevchenko National University “Chernihiv Colehium” (Approval no. 28/09/2020) and the Institutional Review Board of T.H. Shevchenko National University “Chernihiv Colehium” granted for the randomisation process, potential confounding factors, and their control aimed at strengthening the reproducibility of the study. The study complied with Directive 2010/63/EU on the protection of animals used for scientific purposes [65] and the Polish Act of 15 January 2015 on the protection of animals used for scientific or educational purposes (Journal of Laws of 15 January 2015, item 266) [66].

### 4.2. Animals and Experimental Groups

Male Wistar rats (180–220 g) were used in this study. The animals were housed (6 per cage) in a room with a 12:12 h light-dark cycle. Throughout the experimental period, the animals were maintained at a stable temperature of 21 ± 2 °C and had ad libitum access to food and water. Rats’ health and physical activity were monitored daily.

#### 4.2.1. Intermittent Hypoxia (IHT) Protocols

The intermittent hypoxia protocols used in this study were the subject of previous publications and are described in [23,27,31]. The animals were housed in cylindrical Plexiglas chambers (*n* = 6) during the IHT session. The gases were filtered for microparticles and bacteria using high purity compressed air filters. They were humidified to ~30–50% relative humidity and connected to a circulating water bath. A hypobaric chamber was used to expose the rats to intermittent hypoxia (equivalent to 5,000 m altitude, barometric pressure P_B_ = 404 mmHg, PO_2_ = 84 mmHg) for five 30-min cycles per day for 5, 10 and 14 days. Each cycle included a 15-min infusion of a gas mixture containing 12% O_2_, followed by a 15-min infusion of ambient air. The level of the O_2_ fraction in the chambers was monitored throughout the hypoxia protocol using an analyser. Normoxic animals were maintained in the normoxic environment (a continuous gas flow of 21% O_2_) for an equivalent period.

#### 4.2.2. Acute Hypoxia (AH) Protocol

The AH protocol was identical in all the groups studied and consisted of one episode of hypoxia at 7% O_2_ for 30 min.

#### 4.2.3. Acute Emotional Stress Protocol

Stress was modelled using the “free floating in a cage” method [67] for 1 h. The animals swam in a standard plastic cage (50 × 30 × 20 cm) filled with water (22 °C) to a height of 15 cm. The cage was covered with a net at the top. Each cage contained six animals. The distance from the top of the net to the water surface was 5 cm.

The rats were randomly assigned to groups (*n* = 6) within each group of animals as follows: (1) A control group (no treatment), (2) An L-arginine group consisting of rats injected with L-arginine (600 mg/kg body weight, 30 min), (3) An L-NNA (N^ω^-nitro-L-arginine as an inhibitor of NO synthase) group consisting of rats injected with L-NNA (0.35 mg/kg body weight, 30 min), (4) A stress group. A series of acute emotional stress-related animal studies were performed. In accordance with the method proposed by [67], the animals were placed in a cage closed with a net, where the water level up to the net was 5 cm. The duration of the animals’ stay in these conditions was 30 min, (5) An L-arginine + stress group. Prior to the study, the animals were given 1 mL of L-arginine (600 mg/kg body weight, 30 min), (6) An L-NNA + stress group. Prior to the study, the animals were given 1 mL of L-NNA (0.35 mg/kg body weight, 30 min), (7) An IHT group, 5 days. The rats in this group underwent intermittent hypoxic training (IHT) for 5 days. Each day, the rats were placed in a chamber that alternated between 10% oxygen in nitrogen and room air (21% O_2_) at 15-min intervals. The rats received a parenteral injection of 1 mL of normal saline before each hypoxic training session. Five days after the last session of IHT, the rats were sacrificed by decapitation, (8) An IHT group, 10 days. As described above, the rats in this group were subjected to 10 days of IHT. Ten days after the last IHT session, the rats were decapitated, (9) An IHT group, 14 days. As described above, the rats in this group underwent 14 days of IHT. The rats were decapitated 14 days after the last IHT session, (10) An L-arginine + IHT group. The rats received a parenteral injection of 1 mL L-arginine (600 mg/kg body weight, 30 min) prior to each hypoxic training session. Fourteen days after the last IHT session, the rats were decapitated, (11) An L-NNA + IHT group. The rats received a parenteral injection of 1 mL L-NNA (0.35 mg/kg body weight, 30 min) prior to each hypoxic training session. The rats were decapitated 14 days after the last IHT session, (12) An IHT + stress group. After the last IHT session, the rats were subjected to acute emotional stress and then decapitated, (13) An L-arginine + IHT + stress group. The rats received a parenteral injection of 1 mL L-arginine (600 mg/kg body weight, 30 min) prior to each hypoxic training session. After the last IHT session, the rats were subjected to an acute emotional stress test and then decapitated, (14) An L-NNA + IHT+ stress group. The rats received a parenteral injection of 1 mL L-NNA (0.35 mg/kg body weight, 30 min) prior to each hypoxic training session. After the last IHT session, the rats were subjected to an acute emotional stress test. The rats were then decapitated. All the rats were euthanised by intraperitoneal injection of a lethal dose of sodium pentobarbital (Morbital, Biowet, Pulawy, 200 mg/kg body weight).

#### 4.2.4. Samples

Peripheral whole blood samples were collected from the rats by cardiac puncture (under anaesthesia) into 10% EDTA Vacutainer tubes and 3.8% sodium citrate tubes. The samples were then stored at 4 °C until processed (within 8 h). The samples were centrifuged at 3,000 rpm for 10 min to obtain plasma. Rats’ livers were removed immediately after decapitation. In our study, the liver was used for mitochondrial respiration, for the preparation of microsomes and for biochemical analyses. Tissue from a single animal was used for each mitochondrial preparation. The livers were dissected, weighed, and washed in ice-cold buffer containing 2 mM K_2_CO_3_, 10 mM HEPES, and 1 mM EGTA (pH 7.2).

Liver mitochondria were re-suspended in isolation buffer. The mitochondrial suspension (4–6 mg protein/mL) was kept on ice prior to the experiments. A detailed description of the techniques used for mitochondrial isolation and analysis can be found in our previous publication [23].

#### 4.2.5. Assessment of Mitochondrial Respiration Using the Oxygraphic Method

Oxygen uptake was measured using a Clark-type oxygen electrode. The electrode was placed in a magnetically stirred sample chamber (1 mL) in a water bath. The rate of oxygen consumption was quantified as nanograms of oxygen per minute per milligram of mitochondrial protein [35,36,37]. The mitochondria were placed in a respiration chamber containing a mixture of 120 mM KCl, 2 mM K_2_CO_3_, 2 mM KH_2_PO_4_, and 10 mM HEPES, with the pH value maintained at 7.20 at 26 °C using 1.0 N potassium hydroxide. Oxidative substrates included α-ketoglutarate (1 mM final concentration), succinate (0.35 mM), 3 mM glutamate, and ADP (0.2 mM) as a phosphate acceptor. Inhibition analyses using rotenone (10 µM), which inhibits complex I activity in the mitochondrial electron transport chain, and malonate (2 mM), a competitive inhibitor of succinate oxidation by complex II, were performed to assess the role of different substrates in mitochondrial oxidation. A detailed analysis of oxygen consumption parameters, including respiratory rates in states 2, 3, and 4, the V_3_/V_4_ ratio, and the ADP/O ratio, can be found in our previous paper [23].

#### 4.2.6. Microsome Preparations

A liver portion was immediately cooled in ice-cold 1.15% KCl buffer (pH 7.4) for preparation of microsomes. All tissue handling was performed in a cold room. The temperature of the liver was maintained between 0 and 4 °C. Frozen tissues were homogenised in ice-cold homogenisation buffer (1.15% KCl, 0.1M Tris-HCl) at pH 7.4 using a Potter-Elvehjem glass homogeniser fitted with a Teflon pestle. The isolation of microsomes was performed according to the method described by [68]. Homogenised liver was first centrifuged at 16,800× *g* for 20 min at 0–4 °C. The resulting supernatant was centrifuged at 15,000× *g* for 60 min. The cytosol was collected and stored at −80 °C. The microsomal pellet was resuspended in 15 mL of 0.4 M sucrose and 77 mM sodium pyrophosphate (pH 7.5). This was followed by another round of centrifugation in the same conditions. To obtain a protein concentration of approximately 30 mg/mL, the final pellet was resuspended in 150 mM KCl. The protein concentration was quantified with the Bradford method using bovine serum albumin as a reference standard [69].

### 4.3. Biochemical Assays

#### 4.3.1. Assay of Aminopyrine N-Demethylase Activity

A method previously described by [70] was used to measure the activity of cytochrome P_450_-dependent aminopyrine N-demethylase. The assay is based on Nash-formaldehyde detection as described by [71]. The reaction takes place in the presence of cytochrome P_450_, NADPH, and oxygen. 3 mM NADPH, 0.1 M Tris-hydrochloride, 0.25 M Tris, 8 mM aminopyrine, and 5 mM magnesium chloride were used in the reaction mixture during incubation. Hepatic microsomal activity was expressed as nanomoles of formaldehyde produced per minute per milligram of microsomal protein.

#### 4.3.2. 2-Thiobarbituric Acid Reactive Substance (TBARS) Assay

Lipid peroxidation levels were assessed by measuring the concentration of 2-thiobarbituric acid reactive substances (TBARS) using the method described by [72]. This technique is based on the reaction between 2-thiobarbituric acid and malondialdehyde (MDA) or similar substances, which are by-products of lipid peroxidation. In high temperature and acidic conditions, this reaction produces a pink coloured adduct. The intensity of this colour was measured spectrophotometrically at 532 nm. The results were expressed in nanomoles per millilitre (nmol/mL) or per milligram of protein (nmol/mg protein).

#### 4.3.3. Catecholamine Levels

Catecholamine levels, including epinephrine and norepinephrine, were determined with a differential fluorescence method using aluminium and potassium sulphate as described by [73]. In addition, the fluorometric hydroxyindol assay method, based on iodine oxidation and alkaline rearrangement, was adapted from [74] to measure the fluorescence of the final solution at acidic pH. The concentrations of epinephrine and its precursor dopamine (DOPA) were estimated using the method proposed by [75]. All samples were analysed using a Hitachi fluorescence spectrophotometer with an excitation slit of 2 nm, an emission slit of 10 nm, and a sensitivity setting of 1. The fluorescence activation wavelengths were 380–480 nm for norepinephrine and 410–500 nm for epinephrine.

#### 4.3.4. Cholinergic System Activity

The activity of the cholinergic system was assessed by measuring the level of non-mediated acetylcholine (ACh) and the activity of acetylcholinesterase (AChE) in the blood. The technique for determination of ACh was adapted from the method described by [76] and modified by [77]. The AChE activity assay was performed according to [78]. Briefly, 0.2 mL of samples were added to a solution containing 1.0 mM acetylthiocholine (AtCh), 0.1 mM Ellman’s reagent [5,5-dithio-bis-(2-nitrobenzoic acid, DTNB], and 100 mM phosphate buffer (pH 8.0). The results were expressed as micromoles of ACh per minute per millilitre (μmol∙min^−1^∙mL^−1^).

#### 4.3.5. Statistical Analysis

The results are expressed as mean ± SD. All statistical analyses were performed using STATISTICA 13.3 (TIBCO Inc., Palo Alto, CA, USA). To ensure that the number of subjects tested was sufficient to obtain reliable results, a statistical Power Analysis was performed using Statistica. The analysis used tests depending on the test used in the study to compare results between groups. A significance level of 0.05 was assumed and the power of the test was 0.80, which is considered the standard power threshold. The study assumed an expected effect size of 0.5 for the *t*-test or 0.25 for the ANOVA, which is a medium effect size. The results of the analysis showed that the number of six individuals tested in each group was sufficient to achieve the assumed power of the test, confirming the reliability of the results obtained.

We compared groups to assess differences in results between specific study conditions. The rationale for this was to detect any significant differences in results between different experimental conditions. This analysis was carried out separately for the stress group and the IHT (intermittent hypoxic training) group, IHT and stress, as well as for the effects of nitric oxide donors in the context of both stress and IHT. Each of these groups underwent independent analyses to take account of their conditions.

The normality of data distribution was assessed using Kolmogorov-Smirnov and Lilliefors tests (*p* > 0.05). Homogeneity of variance was assessed with Levene’s test. ANOVA, Student’s *t*-test and Mann-Whitney U-test were used to determine differences in the enzyme and substrate levels between the control and experimental groups. The statistical significance level was set at *p* < 0.05. In addition, the relationships between individual data were examined using Spearman correlation analysis.

## 5. Conclusions

In our study of changes in oxygen-dependent mitochondrial energetic processes in the liver when different oxidation substrates, such as succinate (SC), glutamate, and ketoglutarate (KGL), are used in the Krebs cycle, and the analyses of the role of the first and second mitochondrial complexes in oxidative phosphorylation using specific inhibitors under stress and interval hypoxia, we demonstrated an important role of nitric oxide synthase precursors in maintaining NAD-dependent oxidation in liver mitochondria in stress conditions. Our results showed that the stress method used in the study caused a decrease in the efficiency of NAD-dependent oxidation, compared to SC oxidation and depletion of respiratory activity reserves caused by high concentrations of catecholamines. Our data of the interdependence of succinate-dependent oxidation and the functional role of NAD-dependent oxidation in the mitochondrial respiratory chain in stress conditions highlights the importance of the nitric oxide system. The results indicate that the nitric oxide system improves mitochondrial function through L-arginine.

Our data revealed that the administration of L-arginine 30 min before the exposure to stress increased the compensatory capacity of the rat organism. However, the administration of the nitric oxide synthase inhibitor L-NNA prior to the stress did not produce the same changes, indicating opposite effects of L-arginine. Therefore, L-arginine enhanced the compensatory capacity of the sympathoadrenal system under stress, suggesting its potential to mitigate the undesirable effects of stress. In the early stages of IHT, catecholamine concentrations are modulated, reducing lipoperoxidation processes. In the final stages, catecholamine concentrations increase, further reducing lipoperoxidation processes when L-arginine is introduced. Our studies also showed an increase in the blood levels of catecholamines and ACh together with a decrease in the activity of their hydrolysing enzyme at different stages of adaptation to hypoxia achieved by the IHT course (5th, 10th, and 14th day). Thus, the IHT method significantly mobilises the reserve capacities of oxygen-dependent processes via the ACh-AChE system.

## Figures and Tables

**Table 1:** Parameters of the processes of mitochondrial respiration and oxidative phosphorylation in liver mitochondria from rats after administration of L-arginine (600 mg/kg, 30 min) or L-NNA (35 mg/kg, 30 min) in stress-induced conditions. Substrates of oxidation: 0.35 mM succinate and 1 mM α-ketoglutarate (M ± m, *n* = 6).

Group of Animals	V_3_, ng O·min^−1^·mg^−1^ Protein	RCR, V_3_/V_4_	ADP/O, µmol ADP·ng^−1^ O
0.35 mM succinate			
Untreated control group	50.12 ± 3.24	3.04 ± 0.21	1.12 ± 0.06
Stress group	73.58 ± 6.95 *	6.02 ± 0.35 *	1.02 ± 0.01
L-arginine + stress group	39.47 ± 3.52 **	2.75 ± 0.06 **	2.06 ± 0.05 **
L-NNA + stress group	69.91 ± 5.71	4.56 ± 0.58 **	1.06 ± 0.05
1 mM α-ketoglutarate			
Untreated control group	31.12 ± 2.45	3.87 ± 0.08	1.75 ± 0.05
Stress group	44.85 ± 4.48 *	4.71 ± 0.35 *	1.34 ± 0.13 *
L-arginine + stress group	25.98 ± 1.15 **	3.01 ± 0.07 **	0.97 ± 0.04 **
L-NNA + stress group	63.04 ± 2.65 **	2.44 ± 0.07 **	0.99 ± 0.12

Data expressed as mean ± SD (*n* = 6). * Significant differences between the stress group and the untreated control group (*p* < 0.05), ** Significant differences between the stress group and the L-arginine + stress or L-NNA + stress groups (*p* < 0.05).

**Table 2:** Parameters of the processes of mitochondrial respiration and oxidative phosphorylation in liver mitochondria from rats after administration of L-arginine (600 mg/kg, 30 min) or L-NNA (35 mg/kg, 30 min) in stress-induced conditions. Substrates of oxidation: 1 mM α-ketoglutarate + 2 mM malonate, 0.35 mM succinate + 10 μM rotenone, 0.35 mM succinate + 3 mM glutamate + 10 μM rotenone (M ± m, *n* = 6).

Group of Animals	V_3_, ng O·min^−1^·mg^−1^ Protein	RCR, V_3_/V_4_	ADP/O, µmol ADP·ng^−1^ O
1 mM α-ketoglutarate + 2 mM malonate			
Untreated control group	41.82 ± 4.22	3.27 ± 0.24	2.22 ± 0.07
Stress group	44.71 ± 4.78	3.71 ± 0.26	2.23 ± 0.08
L-arginine + stress group	76.29 ± 11.54 **	5.09 ± 0.42 **	2.42 ± 0.04
L-NNA + stress group	32.48 ± 2.71	3.31 ± 0.28	2.28 ± 0.09
0.35 mM succinate + 10 μM rotenone			
Untreated control group	31.12 ± 2.45	3.13 ± 0.18	2.21 ± 0.11
Stress group	48.65 ± 4.48 *	3.69 ± 0.25	1.85 ± 0.08 *
L-arginine + stress group	28.18 ± 1.15 **	4.57 ± 0.30 **	1.78 ± 0.06 **
L-NNA + stress group	34.06 ± 3.65 **	3.25 ± 0.21	2.07 ± 0.12 **
0.35 mM succinate + 3 mM glutamate + 10 μM rotenone			
Untreated control group	46.12 ± 2.97	4.74 ± 0.36	2.55 ± 0.06
Stress group	68.33 ± 7.45 *	3.08 ± 0.17 *	1.79 ± 0.06 *
L-arginine + stress group	52.14 ± 4.45 **	5.98 ± 0.58 **	2.17 ± 0.08 **
L-NNA + stress group	69.41 ± 7.25 **	4.39 ± 0.28 **	2.06 ± 0.08 **

Data expressed as mean ± SD (*n* = 6). * Significant differences between the stress group and the untreated control group (*p* < 0.05), ** Significant differences between the stress group and the L-arginine + stress or L-NNA + stress groups (*p* < 0.05).

**Table 3:** Parameters of the processes of mitochondrial respiration and oxidative phosphorylation in liver mitochondria from IHT-treated rats exposed to stress conditions and after administration of L-arginine (600 mg/kg, 30 min) or L-NNA (35 mg/kg, 30 min) in stress-induced conditions. Substrates of oxidation: 0.35 mM succinate and 1 mM α-ketoglutarate (M ± m, *n* = 6).

Group of Animals	V_3_, ng O·min^−1^·mg^−1^ Protein	RCR, V_3_/V_4_	ADP/O, µmol ADP·ng^−1^ O
0.35 mM succinate			
Untreated control group	50.12 ± 3.24	3.04 ± 0.21	1.12 ± 0.06
IHT and stress group	61.56 ± 5.31	4.67 ± 0.22 *	2.08 ± 0.04 *
IHT, L-arginine + stress group	56.38 ± 5.16	3.44 ± 0.04 **	2.33 ± 0.04 **
IHT, L-NNA + stress group	71.67 ± 9.23	4.56 ± 0.41	2.11 ± 0.05
1 mM α-ketoglutarate			
Untreated control group	31.12 ± 2.45	3.87 ± 0.08	1.75 ± 0.05
IHT and stress group	49.29 ± 3.45 *	4.63 ± 0.41 *	2.34 ± 0.14 *
IHT, L-arginine + stress group	39.78 ± 2.18 **	4.06 ± 0.17	2.97 ± 0.11 **
IHT, L-NNA + stress group	59.19 ± 2.65 **	3.49 ± 0.09 **	2.99 ± 0.32 **

* Significant differences between the stress group and the untreated control group (*p* < 0.05), ** Significant differences between the IHT + stress group and the IHT, L-arginine + stress or IHT, L-NNA + stress groups (*p* < 0.05).

## Data Availability

All relevant data are within the manuscript. Further inquiries can be directed to the corresponding author.
